# Supplementary material for: A Comprehensive Assessment of Ultraviolet-Radiation-Induced Mutations in Flammulina filiformis Using Whole-Genome Resequencing
Source: J Fungi (Basel). 2024 Mar 20;10(3):228. doi: 10.3390/jof10030228 (PMC10971301; doi:10.3390/jof10030228)
Supplement: Supplementary file 1 [file jof-10-00228-s001.zip › Supplementary Material S8/KEGG annotation/out/64381550635650.os/KO/out_map/map04723.html]

KEGG PATHWAY: Retrograde endocannabinoid signaling - Reference pathway


|  |  |
| --- | --- |
| **Retrograde endocannabinoid signaling - Reference pathway** |  |

[
Pathway menu
| Organism menu
| Pathway entry
| Show description
| User data mapping
]

Endogenous cannabinoids (endocannabinoids) serve as retrograde messengers at synapses in various regions of the brain. The family of endocannabinoids includes at least five derivatives of arachidonic acid; the two best characterized are arachydonoyl ethanolamide (anandamide, AEA) and 2-arachydonoil glycerol (2AG). They are released from postsynaptic neurons upon postsynaptic depolarization and/or receptor activation. The released endocannabinoids then activate the CB1 receptors (CB1R) at presynaptic terminals and suppress the release of inhibitory transmitter GABA (depolarization-induced suppression of inhibition, DSI) or excitatory transmitter glutamate (depolarization-induced suppression of excitation, DSE) by inhibiting Ca2+ channels. Whereas DSI and DSE result in short-term synaptic plasticity, endocannabinoids also mediate long-term synaptic changes (eCB-LTD). Persistent activation of CB1 receptors over a period of minutes triggers eCB-LTD by a RIM1alpha-dependent mechanism.

|  |  |
| --- | --- |
| Reference pathway | 100% |
